# A Prospective Monocentric Study of Invasive Breast Carcinoma Diagnosed at 80 Years and Older: Survival Outcomes and Peculiar Challenges

**DOI:** 10.3390/cancers16244142

**Published:** 2024-12-12

**Authors:** Donatella Gambini, Valentina Veronesi, Luca Despini, Stefano Ferrero, Claudia Rossi, Ornella Garrone, Marta Rigoni, Paola Cornelia Maria Muti, Letterio Runza, Elisabetta Kuhn

**Affiliations:** 1 Medical Oncology Unit, Foundation IRCCS Ca’ Granda Ospedale Maggiore Policlinico, 20122 Milan, Italy; d.gambini@casadicuraigea.it (D.G.); ornella.garrone@policlinico.mi.it (O.G.); 2 Department of Biomedical, Surgical and Dental Sciences, University of Milan, 20122 Milan, Italy; valentina.veronesi@unimi.it (V.V.); stefano.ferrero@unimi.it (S.F.); marta.rigoni@unimi.it (M.R.); paola.muti@unimi.it (P.C.M.M.); 3 Senology Unit, Foundation IRCCS Ca’ Granda Ospedale Maggiore Policlinico, 20122 Milan, Italy; luca.despini@policlinico.mi.it (L.D.); claudia.rossi@policlinico.mi.it (C.R.); 4 Pathology Unit, Foundation IRCCS Ca’ Granda Ospedale Maggiore Policlinico, 20122 Milan, Italy; letterio.runza@policlinico.mi.it; 5 IRCCS MultiMedica, 20099 Milan, Italy

**Keywords:** breast cancer, elderly, prospective, outcome, treatment

## Abstract

**Background:** The lengthening of the lifespan led to an increase in breast cancer (BC) diagnosed in very old age, but the treatment recommendations in this patient group usually lack evidence-based practice. We conducted a prospective observational monocentric study specifically targeting patients diagnosed with invasive BC at 80 years of age or older. **Methods:** We enrolled 88 patients consecutively observed for a new BC diagnosis at 80 years or older. The aim was to investigate progression-free (PFS) and overall survival (OS), with a long follow-up period, along with clinico-pathological characteristics of the population. **Results:** At the end of the 5-year follow-up, the estimated OS and PFS probabilities were 82.9% (95% CI: 71.3–95.3%) and 64.0% (95% CI: 51.7–79.2%), respectively. After 8.5 years from the BC diagnosis, 48.9% died. The cause of death was BC in 32.6% of patients, different from BC in 13.9%, and unknown in the remaining. Surgery was performed in 69.3% of the cases and was associated with improved 12-month PFS (*p* < 0.001). Adjuvant systemic therapy and radiotherapy were omitted in 32% and 93% of eligible patients, respectively. A higher rate of metastatic disease at the diagnosis was observed in comparison with data described in younger people, as well as a significantly high rate of drop-out (27.3%). **Conclusions:** Ultra-old patients have a not negligible life expectancy; therefore, the oncologic treatment should be optimal and should adequately fight BC, always considering the quality of life of these frail patients. Future research should focus on developing personalized treatment protocols that incorporate comprehensive geriatric assessments and quality-of-life metrics. Additionally, larger, multicentric studies are needed to validate our findings and explore the role of emerging therapies in this age group.

## Simple Summary

The global aging trend led to an increase in cancer diagnoses at a very old age, including breast cancer (BC). The BC treatment recommendations are based on clinical trials that do not include elderly patients, which still miss dedicated guidelines derived from prospective studies. To gain new insight, we performed a prospective monocentric study focused on the characteristics and outcomes of patients aged 80 years or older with a BC diagnosis. Most patients (84.1%) did not have advanced disease, and 69.3% were surgically treated. Systemic therapy was administered to 81.8%. Postsurgical therapy was omitted in 32% of eligible patients due to either comorbidities or patient choice. At the end of the 5-year follow-up period, 25.6% of patients experienced disease progression and the overall survival probability was 82.9. After 8.5 years, 48.9% of patients died, and BC was the cause of death in 32.6% of them. Many patients were lost to follow-up (27.3%). Our results emphasize the importance of tailored management strategies, considering both the biological behavior of BC in elderly patients and their overall health status since their life expectancy is not negligible.

## 1. Introduction

Breast cancer (BC) is the most prevalent cancer among women globally, being the most frequently diagnosed in 157 out of 185 countries. Worldwide, approximately 1 in 8 women (13%) will be diagnosed with BC in her lifetime, and BC incidence escalates with age, peaking in the seventh decade of life [1].

According to the World Health Organization (WHO), in 2022, BC was the second most commonly diagnosed cancer globally, after lung cancer, with more than 2.3 million new cases and accounting for 11.6% of all cancers [2]. In women, BC represents by far the most frequent malignancy, accounting for approximately one fourth of cancer cases and one sixth of cancer deaths worldwide [2]. Notably, the BC age-standardized incidence rate in women worldwide was 46.8% in 2022 and is projected to increase to 48.1% by 2030 [2,3]. The burden of BC in women generally rises with age, with the highest burden expected in the 45–49 age group from 2020 to 2030 [3]. Coherently, older people will also be affected by a general expected increase in incidence.

In the US, the median age of a BC diagnosis for women was 63 years from 2017 to 2021, with some variation based on ethnicity [4]. In Europe, half of all estimated BC cases in women occur in the age group between 45 and 69 years, overlapping with the recommended age range of mammographic screening and emphasizing its clinical relevance [5].

Based on a recent study, the total prevalence of BC in Italian women was 806,411 cases in 2018 [6]. Of these, 25,966 cases were among women aged 0–44 years, 472,461 cases in the 45–74 age group, and 307,983 cases were in women aged 75 years and older. The prevalence proportion in the older group was 7355 per 100,000. In Italy, the estimated total BC prevalence for 2030 is projected to reach 1,053,633 cases, with a concurrent increase among older patients. This expected rise underscores the need for targeted healthcare policies and personalized treatment strategies to address the specific needs of the aging population, particularly those with comorbidities and other vulnerabilities. Previous studies showed an important role of age at the diagnosis in the BC prognosis and a likely more aggressive biological behavior of BC in younger patients [7,8].

Nevertheless, most research data on BC is derived from clinical studies where younger individuals are more prominently represented, and with the high prevalence of BC in patients older than 70 years, there is a growing concern about the necessity of studies focusing on elderly patients. Elderly people represent a not quantitatively negligible cohort of patients, for whom the therapeutic index is often very difficult to establish. Older people are frail due to both age and comorbidities, but also due to lower compliance with treatment and more frequent drop-outs from treatment and controls. In recent years, many retrospective analyses and systematic reviews have been performed, considering women aged 65 and older, typically extending the cut-off to 70 or 75, and in some instances, surpassing 80 years [9,10].

Notably, only a few studies specifically address the age group of 80 years and older, and the current landscape and future perspectives for geriatric BC are mainly derived from studies focusing on patients older than 60–65 years [11,12,13].

Recently, with growing awareness of this issue, specific recommendations have been developed and promoted by dedicated scientific societies, such as the European Society of Breast Cancer Specialists (EUSOMA), and the International Society of Geriatric Oncology (SIOG). These guidelines are regularly updated and address various aspects related to the management of elderly BC patients. They detail the usefulness of different therapeutic options, employing an evidence-based approach tailored to the elderly population, often defined as those over 70 years of age. When available, these guidelines also consider specific age thresholds to guide clinical decision making [14]. Importantly, evaluating competing mortality risks plays a crucial role in the decision-making process to prevent unnecessary toxicities. The use of specific predictive tools is recommended in order to take into account both life expectancy and cancer risk. For instance, the National Comprehensive Cancer Network (NCCN) and the American Society of Clinical Oncology (ASCO) guidelines endorse the use of ePrognosis (https://eprognosis.ucsf.edu/). Similarly, EUSOMA and SIOG recommend tools such as Predict (https://breast.predict.cam), and the Age Gap Decision Tool (https://agegap.shef.ac.uk) [15,16,17].

In an effort to obtain more reliable data and gain insights on the characteristics and outcomes of BC diagnosed in patients aged 80 years and older, we conducted a prospective monocentric study specifically targeting this age group, which has previously been studied primarily through retrospective analyses. The aim was to investigate progression-free survival (PFS) and overall survival (OS) over a 5-year period, with OS monitored during an extended follow-up. Additionally, the study describes clinico-pathological characteristics, treatment strategies, and follow-up outcomes.

## 2. Materials and Methods

### 2.1. Patients and Data Collection

From June 2014 to May 2017, all patients aged 80 years and older, both females and males, consecutively observed in the Breast Unit of the Fondazione IRCCS Ca’ Granda Ospedale Maggiore Policlinico for a new histological diagnosis of BC, were eligible for enrollment. Exclusion criteria included patients observed solely for consultation purposes or patients with a declared intention to transfer to another hospital for follow-up after the initial visit. An electronic database was developed to systematically collect and manage all pre-agreed data, ensuring consistency and accuracy in data handling. We recorded familial and personal history; and clinico-pathological baseline data, including the diagnosis age, pharmacotherapy, clinical stage, pathologic stage, histotype, grading, lymphovascular invasion [18], conventional biological markers (estrogen receptor [ER], progesterone receptor [PR], HER2 status, and ki-67 index), and molecular type. Tumor histotype, grade, and stage were assigned according to the WHO classification, the Elston–Ellis grading system, and the eighth AJCC staging system, respectively [19,20,21]. IHC markers for ER, PR, HER2, and ki-67 were categorized according to the criteria of the 2011 St. Gallen International Breast Cancer Conference, as well as the molecular classification, which was based on surrogate definitions by means of IHC markers [22]. A geriatric evaluation was conducted and recorded using the geriatric G8 (categorized as <14 or ≥14) and the Charlson Comorbidity Score (CCI, categorized as 0–1 and ≥2) [23,24]. Therapies and outcomes were assessed at different times of follow-up, usually checked biannually.

The progression of disease was defined according to RECIST criteria [25]. The Predict tool was used to estimate the expected benefits of adjuvant therapy.

Primary endpoints were PFS and OS. Secondary aims included the (a) description of clinico-pathological characteristics of ultra-old women with a new diagnosis of BC; (b) identification of causes of death, when available; and (c) assessment of drop-out rates and their causes, when known. Drop-out was defined as the cessation of follow-up for known or unknown reasons, different from death. The planned follow-up period was set to extend until May 2019 (five years from the first enrollment). For all patients (including those lost to follow-up before May 2019), the date and cause of death were verified by phone request to relatives or, when not possible, through a regional electronic system (available only for patients who died in the same administrative region as their official residence) after 8.5 years from the first enrollment.

This study received ethical approval from the local ethical committee (number 25/2014), and all patients signed specific informed consent.

### 2.2. Statistical Analysis

Data were reported as the mean and standard deviation (SD), median and interquartile range (IQR), and minimum–maximum range for interval/ratio scale variables and as absolute and relative frequencies for categorical variables.

Considering the planned follow-up period (5 years), OS and PFS were obtained by means of Kaplan–Meier (KM) estimation. For OS, death from any cause was treated as the event, while progressive disease (PD) was ignored. For PFS, both PD and death from any cause were treated as events, considering the time until the first occurrence of either one. KM estimates for OS and PFS were calculated for the entire sample and within subgroups defined by age at the diagnosis (≤82.5 versus >82.5 years; the cut-off was determined using the receiver operating characteristics’ curve methodology considering the first event that occurred), the G8 score (<14 versus ≥14), the CCI score (0–1 versus ≥2), and whether surgery was performed (yes versus no). Between groups, survival curves were compared by means of the log-rank test.

To further investigate the risks of PD and death, cumulative incidence curves for each competing event were calculated using the Aalen–Johansen estimator, providing crude incidence rates that account for the competing nature of death on PD. The Grey test was used to compare cumulative incidence curves between subgroups.

The cumulative incidence of BC-related death was examined separately from PD, considering death from other causes as a competing risk, using the Aalen–Johansen estimator. This analysis included data collected both directly during the study and from regional registries covering the entire follow-up period. This approach improves the precision of mortality estimates and mitigates potential underestimates associated with drop-outs observed during the planned follow-up period. Because data on the cause of death (neoplasm versus other cause) were only available for a subset of patients (47% of those who experienced the event), two analyses were performed to define a lower and an upper bound for the crude incidence curves. Specifically, the lower bound was obtained by assigning “death from another cause” to patients with an unknown cause of death, while the upper one was obtained by considering mortality for a neoplasm in patients with an unknown cause of death.

All statistical analyses were performed using R software, version 4.1.2. [26]. *p*-Values are two-sided, and the significance level was defined as *p* < 0.05 for the main analyses and *p* < 0.005 for subgroup analyses, according to Bonferroni’s correction.

## 3. Results

### 3.1. Patient Description

A total of 88 patients were included in this study, all female, except for two (86, 97.7%). Patient characteristics are detailed in Table 1.

The average age at the diagnosis was 84.5 years, and a large majority of patients (81.8%) were still independent. Among the 88 patients, 24 (27.3%) sought medical care with advanced signs of disease. We defined “delayed access” as cases where patients had an evident palpable mass, a T stage of 3 or higher, and/or skin involvement, such as ulcers or visible skin retraction. Additionally, 14 patients (15.9%) were diagnosed with metastatic breast cancer at the time of presentation.

Regarding medications, antihypertensive drugs were the most commonly used, taken by 73.9% of patients. Additionally, 45.4% were on antiplatelet or anticoagulant therapy, and 32.9% used antiresorptive medications, which was considered a substantial use. Polypharmacy, defined as the regular use of five or more drugs, was observed in 60.7% of patients.

Regarding the suspicion of genetic predisposition, familial history data were available for 83 patients, of whom 26 (31.3%) had at least one first-degree relative diagnosed with BC. In terms of personal history, 16 patients (18.2%) had a previous (metachronous) BC (in situ or infiltrative), while 2 patients (2.3%) had synchronous BC. Among the 16 patients with metachronous BC, recurrence could be excluded in 10 cases (62.5%) based on differences in biological factors or tumor laterality. However, in 6 cases (37.5%), recurrence could not be definitively ruled out.

For those with previous BC, the median age at the first diagnosis was 76 years (range: 43–88) or 74 years (range: 43–82) when excluding cases with possible recurrence. None of the patients with synchronous neoplasms had a lobular histotype. During follow-up, one patient developed subsequent metachronous BC.

Seventeen patients (19.3%) had a history of 17 different cancers before the BC diagnosis. Among these, only two had previously received systemic chemotherapy (both for lymphoma, one of whom was treated with an anthracycline-based regimen), and one patient was treated with radioiodine for a benign thyroid condition. During follow-up for BC, one patient developed renal cancer, which was treated with radiofrequency ablation (Table 1).

### 3.2. Tumor Characteristics and Oncological Therapies

A total of 91 BCs were diagnosed, as 2 patients showed synchronous neoplasms and 1 patient developed a subsequent metachronous neoplasm. Tumor characteristics are detailed in Table 2.

Seventy-four patients were diagnosed with 77 non-advanced BCs; among them, surgery was the most common treatment option following the diagnosis. Fifty-four lesions (70.1%) were initially treated surgically, with an additional four cases (5.4%) undergoing surgery after neoadjuvant therapy. Of these, two patients received endocrine therapy (ET) and two received chemotherapy. The median age of patients who underwent surgery as the first treatment was 83 years (range: 80–93), while the median age of those treated primarily with systemic therapy—almost exclusively ET—was 87 years (range: 82–101). The decision to use ET as the primary therapy rather than surgery was influenced by factors such as older age, patient preference, tumor size, and/or severe comorbidities.

In cases where surgery was performed, a concomitant axillary procedure was carried out in only 33 cases (36.3%). When considering the entire cohort, including advanced BC cases, surgical intervention was performed in 69.2% of patients.

Tumor laterality was left-sided in 49 patients (53.8%) and right-sided in 42 patients (46.2%). Among the removed BCs, the median tumor size was 1.8 cm (range: 0.4–10.5 cm). Histologically, 55 tumors (60.4%) were classified as no special type, 15 (16.5%) as lobular, 10 (11.0%) as mucinous, 3 (3.3%) as mixed, and 8 (8.8%) as other special types. These included four apocrine, three papillary, and one metaplastic carcinoma. Of the 91 BCs, 8 (8.8%) were grade 1, 53 (58.2%) were grade 2, and 30 (33.0%) were grade 3. The molecular intrinsic subtypes were luminal A in 38 cases (41.8%), luminal B in 37 cases (40.7%), luminal B–HER2-positive in 7 cases (7.7%), triple-negative in 7 cases (7.7%), and HER2-positive in 2 cases (2.2%).

Adjuvant radiotherapy (RT) was administered to only 6 patients (6.8%) out of 50 eligible patients. Eligibility was defined as having undergone quadrantectomy, no prior radiation treatment to the same breast, and no absolute contraindications. The main reasons for omitting RT were comorbidities, advanced age, and patient preferences. The median age of the patients who received RT was 83 years (range: 80–84).

Regarding systemic therapy, 72 patients (81.8%) received treatment, including 1 patient diagnosed with two metachronous BCs during the study period. In the non-advanced setting, ET was prescribed as primary or adjuvant treatment for 56 patients (75.7%), while only 2 patients (2.3%) received chemotherapy due to ER/PR negativity. Seventeen of the fifty-three patients (32%) did not receive any adjuvant therapy due to factors such as advanced age, patient preferences, comorbidities, and/or an unfavorable therapeutic index. The median age of this group was 83 years (range: 80–93). Interestingly, most patients who did not receive adjuvant therapy had luminal A BC, which is associated with a very good prognosis (11/17, median age: 80 years, range: 80–93). The remaining six patients had either triple-negative (TN) or luminal B–HER2-positive BC (median age: 84.5 years, range: 81–90). Among these 17 patients, only 1 experienced disease progression (Table 3). None of the eight patients (10.1%) with HER2-positive BC received adjuvant trastuzumab, primarily due to age, comorbidities, and patient preferences. The median age of this cohort was 85.5 years (range: 81–101).

### 3.3. Toxicity

Regarding toxicity and serious adverse events during follow-up, one case of severe pulmonary thromboembolism occurred in an 80-year-old patient during the first months of letrozole therapy. This patient was also diagnosed with significant dyslipidemia concurrently with breast cancer, which was promptly treated with statins, since it represented a potential risk factor. Additionally, accidental bone fractures were reported in three patients aged 93, 86, and 82, occurring during no therapy, tamoxifen therapy, and aromatase inhibitor therapy, respectively. Other serious events registered included a cardioembolic stroke due to atrial fibrillation in an 82-year-old patient treated with an aromatase inhibitor and a myocardial infarction in an 81-year-old patient, also treated with an aromatase inhibitor, who subsequently developed normotensive hydrocephalus. Cognitive decline was observed in three patients aged 81, 82, and 85, respectively, with two of them receiving adjuvant ET. Given the various discomforts and symptoms already reported by patients before starting oncological therapies, we chose to document only more severe adverse events (grade 3–4 toxicities according to Common Terminology Criteria for Adverse Events (CTCAE) v5.0) to ensure the reliability of the data [27]. ET was discontinued in two patients (2.3%) due to intolerance: one case of letrozole-related osteoarthralgias and one case of tamoxifen-related recurrent vaginitis (in a hysterectomized patient with osteoporosis where tamoxifen was preferred).

### 3.4. Drop-Outs

Under the competing risks’ framework, which accounted for both PD and death, 24 participants (27.3%) were lost to follow-up before experiencing an event. When only death was considered, the number of drop-outs increased to 35 (39.8%). Patients were lost to follow-up for various reasons, including transfer to nursing homes, relocation to another center or administrative region, or further clinical decline not necessarily related to oncological reasons, which often led to the initiation of home care. These categories sometimes overlapped; for example, a patient might be transferred to a nursing home due to clinical decline. Notably, eight patients were lost to follow-up after experiencing PD, including one who was transferred to a nursing home and two who relocated out of the region.

### 3.5. Progression-Free and Overall Survivals During the Planned Follow-Up Period

**Figure 1:**
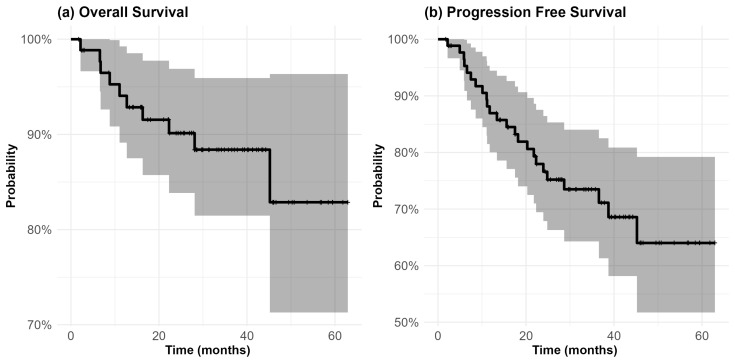
Kaplan–Meier curves (solid lines) for overall survival (**a**) and progression-free survival (**b**) over a 5-year (60 months) follow-up period. Gray-shaded areas represent the 95% confidence intervals for each survival estimate, while vertical segments (|) represent censored patients.

During the planned follow-up period, 10 patients (11.4%) died from any cause, with half of these deaths (5 patients) occurring after PD. The median time from PD to death among these patients was 2.8 months (Q1–Q3: 1.5–4.7 months). The KM-estimated OS probability, regardless of PD status or cause of death, was 94.1% (95% CI: 89.1–99.2%) at 12 months, 88.4% (95% CI: 81.5–95.9%) at 30 months, and 82.9% (95% CI: 71.3–96.3%) at 60 months from the diagnosis (Figure 1a).

Nineteen patients (25.6%) experienced PD during the 5-year follow-up, with a median time to progression of 15.6 months (Q1–Q3: 9.3–22.9 months) from the diagnosis. KM-estimated PFS probabilities are shown in Figure 1b, with rates of 86.9% (95% CI: 80.0–94.4%) at 12 months, 73.5% (95% CI: 64.3–84.0%) at 30 months, and 64.0% (95% CI: 51.7–79.2%) at 60 months.

**Figure 2:**
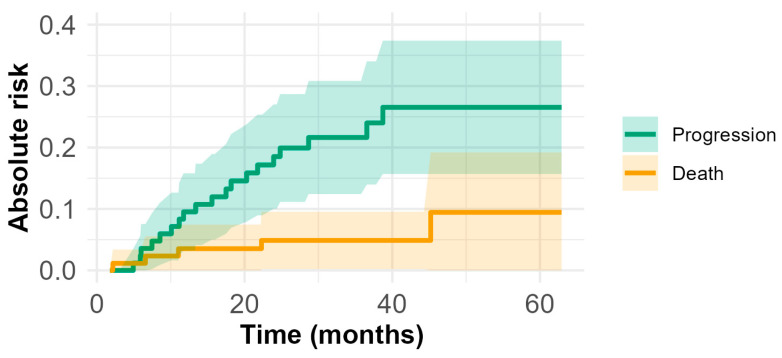
Aalen–Johansen crude incidence estimates of PD and death.

Figure 2 presents the cumulative incidence functions (CIFs) for PD and death, calculated using the Aalen–Johansen estimator. At 30 months, the cumulative incidence rates were 21.6% (95% CI: 12.4–30.8%) for PD and 4.9% (95% CI: 0.2–9.6%) for death. By 60 months, these rates increased to 25.5% (95% CI: 15.7–37.4%) for PD and 9.5% (95% CI: 0–19.2%) for death.

**Figure 3:**
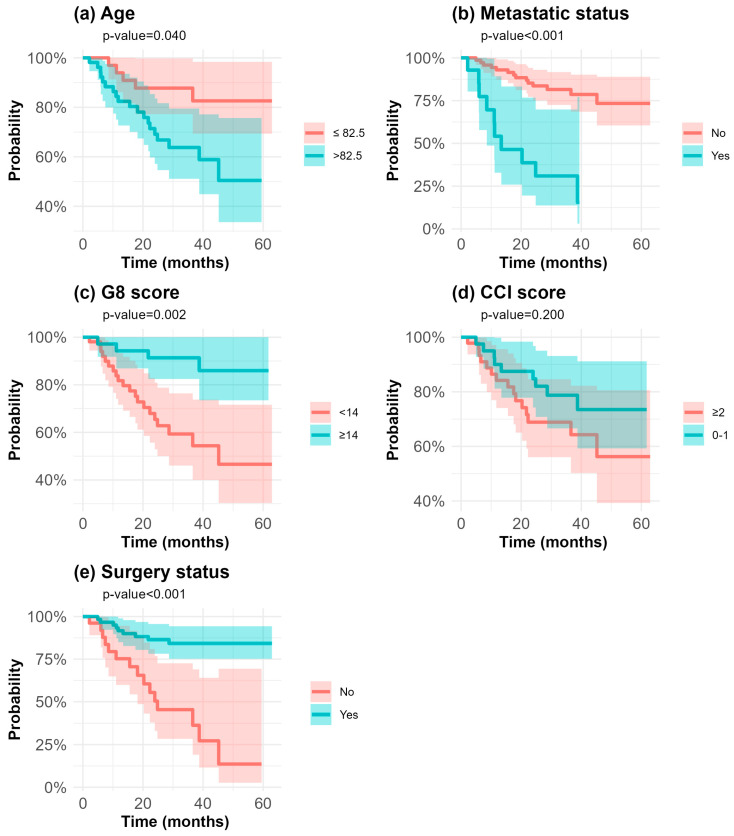
Aalen–Johansen crude incidence curves showing progression-free survival by age (**a**), metastatic status at diagnosis (**b**), G8 score (**c**), CCI score (**d**), and surgery (**e**). Shaded areas around each curve represent 95% confidence intervals associated with curves.

The KM-estimated OS and PFS curves indicate a high probability of survival at 12 months. By 5 years, approximately 83% of patients were still alive, while 64% were both alive and progression-free. The CIFs obtained using the Aalen–Johansen estimator, providing event-specific risks, revealed that by 5 years, PD had occurred in 25.5% of patients, while the competing risk of death accounted for 9.5%. Figure 3 shows the PFS curves stratified by age, metastatic status at the diagnosis, the G8 score, the CCI score, and surgery. Among patients who experienced PD, 42.1% (8 out of 19) were metastatic at the diagnosis. At 12 months, the estimated PFS rates were 54.2% (95% CI: 32.9–89.3%) for metastatic patients and 93.0% (95% CI: 87.2–99.1%) for non-metastatic patients at the diagnosis (log-rank test, *p* < 0.001). Of note, among non-metastatic patients, among the 14 patients who experienced either PD or death before progression, 6 had undergone surgery. A total of 61 patients underwent surgery. At 12 months, the estimated PFS rates were 91.7% (95% CI: 84.9–98.9%) for those who underwent surgery and 75.3% (95% CI: 59.9–94.6%) for those who did not (log-rank test, *p* < 0.001). Within the subgroup of patients experiencing PD, 21.1% (4 out of 19) had a G8 score ≥14, whereas all patients who died before PD had a G8 < 14. At 12 months, the estimated PFS for patients with G8 < 14 was 81.7% (95% CI: 71.6–93.3%), compared to 94.3% (95% CI: 86.1–100%) for those with G8 ≥ 14 (log-rank test, *p*-value = 0.002).

Older patients (>82.5 years) accounted for the majority of progression or death events during the follow-up period (73.7%). At 12 months, the estimated PFS for older patients was 82.4% (95% CI: 72.7–93.6%) compared to 93.9% (95% CI: 86.1–100%) for younger patients (log-rank test, *p*-value = 0.04). For patients with a CCI score ≥2, the absolute number of PD or death events was similar to those with CCI of 0–1 (11 versus 8 events, respectively). At 12 months, the estimated PFS for patients with CCI ≥ 2 was 84.2% (95% CI: 74.1–95.7%) and 90.0% (95% CI: 81.2–99.8%) for patients with CCI at 0–1. Within the considered study cohort of elderly BC patients, those without metastatic disease, those with higher G8 scores (≥14), and those who underwent surgery had a greater likelihood of survival and being free from PD over the 5-year follow-up period.

### 3.6. Overall Survival and BC-Caused Death in the Extended Follow-Up Period (8.5 Years)

Over the entire 8.5-year observation period, which included data obtained from regional registries, a total of 43 deaths (48.9%) were recorded. Among the patients who were still alive (excluding those without registry data), the median age was 90.5 years (interquartile range: 89.5–92.5). Notably, 16 of these deaths occurred within the 5-year planned follow-up period but were identified exclusively through registry data, as these patients withdrew from the study prior to their death. Adding these to the 10 deaths observed among patients who remained in the study provides a more accurate assessment of mortality within the follow-up period. The high drop-out rate would otherwise result in a significant overestimation of 5-year OS. Specifically, the estimated 5-year survival was 82.9% (95% CI: 70.4–95.4%) when considering only patients who completed the follow-up. However, when drop-outs were accounted for, the estimated OS dropped significantly to 58.0% (95% CI: 43.6–77.1%), underscoring the considerable impact of drop-outs on survival estimates.

**Figure 4:**
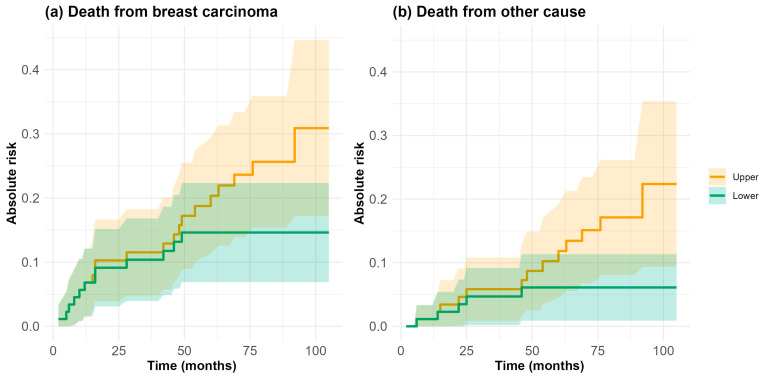
Lower (green) and upper (orange) bounds for crude incidence curves of the two competing risk events: death from breast carcinoma (**a**) and death from another cause (**b**), estimated by means of the Aalen–Johansen estimator. The shaded areas around each curve represent the 95% confidence intervals.

Figure 4 illustrates the lower and upper bounds for the cumulative incidence of death from a neoplasm, treating death from other causes as a competing risk, over the full 8.5-year observation period. At 5 years, the lower bound for the cumulative incidence of neoplasm-related death was 16.1% (95% CI: 8.4–23.9%), assuming that all deaths of unknown cause were attributed to other causes. Conversely, the upper bound was 36.4% (95% CI: 26.1–46.6%), assuming that all unknown-cause deaths were due to a neoplasm. These bounds highlight the potential range for neoplasm-related mortality and highlight the uncertainty introduced by missing information.

### 3.7. Impact of Male Participants on Survival Estimates

The analysis was refined by excluding the two male participants to prevent any potential bias arising from biological differences in breast cancer between males and females. Both male participants, aged 84 and 88 years, died (one from BC and the other from unrelated causes). However, they were lost to follow-up during the planned follow-up period (approximately 6 and 15 months after diagnosis), and their survival data were retrospectively retrieved from registry records. As a result, excluding these two participants and re-evaluating OS and PFS using only the study data did not lead to substantial differences in the estimates during the planned follow-up period, since their contribution to the analysis was limited to the group at risk until the time of their loss to follow-up (Figure S1). The OS estimates, obtained with study data, at 12, 30, and 60 months were 94.0% (95% CI: 89.0–99.2%), 88.3% (95% CI: 81.3–95.9%), and 82.8% (95% CI: 71.2–96.3%), respectively. Similarly, the probabilities of being progression-free at 12, 30, and 60 months were 86.8% (95% CI: 79.8–94.4%), 73.3% (95% CI: 64.1–83.9%), and 63.9% (95% CI: 51.6–79.1%). Conversely, when registry data were used to extend the follow-up period, excluding the male participants resulted in slightly greater, though still comparable, changes to the OS estimates. At 5 years, the OS estimate for the female-only group was 59.3% (95% CI: 44.7–78.7%).

## 4. Discussion

The aging trend is becoming more widespread globally, and a rise in BC burden has been recently occurring. The lengthening of the lifespan led to an increase in BC diagnoses at a very old age, and the probability of developing BC in a cancer-free woman over 84 years old is 1 in 38 (2.6%) [1]. For this reason, greater interest has emerged in developing appropriate therapeutic practices for these patients. The challenges related to planning not only diagnostic but also therapeutic strategies for very old people are varied. These challenges are not only due to comorbidities or physical weakness but also to a more complex vulnerability arising from social, familial, and logistical issues.

The crucial aspect of cancer in older people is how and how significantly such a diagnosis can impact survival. Competing death risks may challenge the assessment of OS in these patients. Additionally, while many elderly BC patients may die from causes other than cancer, it is estimated that approximately 40% of women newly diagnosed with BC after 80 years of age will eventually die from this disease [9]. Closely related to this is the therapeutic index of various treatment options available, ranging from surgery to radiotherapy, and how these therapies could affect not only survival but also the quality of life. Moreover, older women with BC tend to receive less aggressive treatment than younger women with comparable diseases, which causes a significant excess in the death rate [28,29].

### 4.1. Older Patients and Clinical Trials

As previously mentioned, data on very elderly populations, particularly those over 80, remain scarce, leading to significant underrepresentation. In contrast, this age group is increasingly prevalent in the real world, and its presence is expected to grow in the future. Actually, elderly patients are generally underrepresented in clinical trials. A recent meta-analysis by Sedrak et al. investigated the difficulties in the inclusion of older patients in clinical trials and the interventions that could promote their participation [30]. In 85% of the studies analyzed, the minimum age used to define older adults was 65 years. The authors highlighted the various barriers to enrolling older patients and provided recommendations to enhance their participation. They acknowledged the challenges in bridging the evidence gap in geriatric oncology but suggested key strategies, such as designing dedicated prospective studies and expanding the use of real-world data. This approach could facilitate retrospective analyses of larger datasets derived from multiple population-based observational cohorts [30].

A recently published interventional trial, including older BC patients, is a prospective phase 2 study evaluating the safety and tolerability of palbociclib in patients aged 70 years and older. The median age was 74 years (range: 70–87). The analyses were also split into two age groups: younger than and older than 75 years. In the latter cohort, higher rates of adverse events and consequent early palbociclib discontinuations were observed [31].

A recent meta-analysis compared the reduction in mortality risk in younger versus older patients (>65 years) treated with cyclin-dependent kinase 4/6 (CDK4/6) inhibitors and revealed a comparable mortality risk reduction of approximately 20% in both age groups [32]. This analysis, the first large, pooled assessment in this context, confirmed that CDK4/6 inhibitors provide significant PFS and OS benefits in patients aged 65 and older. However, no specific analysis was conducted for the ultra-elderly subgroup.

Moreover, a large, prospective multicentric study focused on surgical therapies in BC patients over 70 years old [33]. Although the median age was 76, the authors reported surgical interventions across various age groups, including those aged 80 and older. As the study aimed to evaluate the outcomes of surgical therapy, metastatic patients were excluded. The authors provided data on the safety of surgical options, noting no associated mortality but highlighting a negative impact of surgical treatment on quality of life and functional independence. Several studies have aimed to describe the allocations, efficacy, and toxicities of therapies, as well as survival outcomes in the elderly population. However, most of these studies defined older groups as those aged 65 to 75 years [34]. This is partly due to the difficulty in obtaining reliable statistical data for smaller sample sizes as age increases. Pesavento et al. conducted a pilot study to assess whether a decision aid could address patient-level determinants of low-value treatments in older patients, using a threshold of 70 years to define elderly [35]. Similarly, the GERONTE trial, which enrolled more than 700 elderly cancer patients across Europe, defined “older” as 70 years and above [36].

Some retrospective studies have analyzed patients under 80 years but included subgroup analyses for those older than 80. For instance, Galy et al. concluded that the information from sentinel lymph node biopsies in patients over 80 was not clinically useful as it did not influence subsequent management [37]. Another study by Houvenaeghel et al. on triple-negative BC in the elderly found worse recurrence-free survival, PFS, and OS in patients over 80 compared to younger groups [38]. Additionally, Lodi et al. conducted a systematic review of BC in older patients, comparing patients aged 70–79 with those 80 and older. They found larger tumor sizes, lower LVI, and greater hormone sensitivity in the older group, along with more frequent lymph node involvement, distant metastases, and a higher BC-specific mortality rate, at both 5 and 10 years [9].

### 4.2. Characteristics of BC Diagnosed in the Elderly

During our prospective study on very old people who accessed our Breast Unit for a new diagnosis of BC, we enrolled 88 patients in 3 years, corresponding to approximately 6% of the total number of patients observed for BC in the 2014–2016 triennium in our institution. From 2017 to date, the proportion of women over 80 years old with BC has been slightly more than 10%, and it increases to about 25% when considering people older than 70 years.

When we compared our results with those from larger studies, including old patients even if with lower thresholds for defining older patients [33,39], we found similar ER positivity (90% in our study compared to 85% and 87%) and histological grade (55% grade 2 and 31% grade 3 compared to 54% and 30%, respectively) [33]. Histologically, in our study, 59.5% of BCs were not a special type as compared with 66.8% in the AIRTUM study [39]. Moreover, in our cohort, 10% of BCs were HER2-positive, which is variably lower compared to other studies, approximately 12% reported by two studies and 21% reported in the AIRTUM study [33,39,40]. However, the last study is characterized by a not negligible rate of missing data that could affect the results [39].

In our study, the proportion of patients with stage IV disease was 15.9% compared to 5.8% in the AIRTUM study. This discrepancy may be explained by the older age of our patients, as older individuals, particularly those with comorbidities, may experience delayed access to care, leading to a higher rate of metastatic disease. Coherently, the AIRTUM study also showed an increase in metastatic patients with age.

Similarly, the percentage of patients with distant metastases in our study was more than double (15.9% vs. 5.9%) compared to the data from the systematic review by Lodi et al. [9]. Our cohort also had a higher proportion of G2 tumors (57.2% vs. 45%), ER-positive BCs (90% vs. 77%), and LVI (31.7% vs. 23%). The percentage of no special-type carcinomas was nearly identical, at about 60%. Notably, our study included complete histopathological data, while the systematic review reported 10% of cases with an unknown grade.

In conclusion, since our cohort comprises older patients than those in other studies, we can reasonably assume that BC is more frequently diagnosed at stage IV (metastatic) in older patients compared to younger populations.

### 4.3. Treatment and Follow-Up of Older BC Patients

Treating and monitoring older BC patients present unique challenges due to the scarceness and heterogeneity of this population. Older patients often have multiple comorbidities, functional limitations, and varying degrees of frailty, which can influence treatment choices and outcomes.

When designing studies involving very old populations, significant challenges in complete data collection are anticipated due to the high likelihood of substantial changes in various factors over time. These changes can result in reduced compliance and impact the feasibility of continuing therapies and follow-up assessments. The elevated risk of discontinuation from specialist outpatient services or routine monitoring is a critical factor to consider when planning therapeutic programs. Ensuring strategies to address these potential disruptions is essential for maintaining data integrity and delivering effective care tailored to this vulnerable population. A recent retrospective study focused on Chinese women aged 70 years and older with BC and aimed to develop nomograms for the individual prediction of disease-free survival (DFS) and OS. The study reported a 5-year OS rate of 85.6% and a 5-year DFS rate of 80.1%. Notably, they described a low rate of drop-outs (10.3%) compared with our findings [41].

Another crucial issue in elderly patients concerns adherence to oral therapies. In an earlier analysis using the SEER (Surveillance, Epidemiology, and End Results Program) database, which included cases of stage I–III ER-positive BC patients collected over 15 years (2001–2015), 441 out of 552 women were aged 80 years or older, all treated with primary ET. The study concluded that adherence to therapy was not significantly correlated with a reduction in the risk of death from breast cancer. However, this finding was considered partially influenced by bias, as women with larger tumors might have demonstrated better compliance with therapy.

Our study offers some interesting food for thought. Firstly, many patients access care with some delay; about 25% of patients had a clinically well-appreciable lesion, sometimes ulcerated. While exclusion from dedicated screening is undoubtedly correct for these patients, better information, together with a specific question about breasts or a breast examination during other medical visits, could potentially anticipate the diagnosis and allow less invasive surgery, avoiding further risks of therapy-related toxicities. Secondly, the great preponderance of endocrine-sensitive neoplasms makes such diseases easier to treat, even in the presence of comorbidities. This fact could sometimes allow the omission of surgical procedures, at least as the first option of treatment for older or more frail patients.

Nevertheless, when surgical intervention is indicated, omitting adjuvant therapy, in the presence of favorable prognostic factors, could be a rational option in very old patients. For this group, we calculated the additional benefit from systemic therapy and estimated OS using the Predict tool. In the vast majority of our patients, we found no significant advantage. However, this tool is not indicated for women older than 85 years. The decision whether to start therapy or not was always made in consultation with the patient and/or their relatives.

The main concerns in the oncologic decision-making process regard the risk of under- or overtreatment, particularly for older patients. Of note, a very interesting issue is the potential omission of upfront surgery in patients with larger neoplasms in order to avoid heavier surgical treatments, especially in the presence of comorbidities. In our study, undergoing surgery appears to further improve the chances of remaining progression-free and alive over time in this population. This insight underscores the importance of considering both general health and the potential benefits of surgery in the treatment strategy for older adults with BC.

On the other hand, according to the guidelines, adjuvant radiotherapy as well as systemic therapy, such as ET, should be guided by the expected advantage; their omission is suggested when the possible toxicities outnumber it. In particular, older women are more often affected by arthrosis, osteoporosis, and joint pain, and an aromatase inhibitor could exacerbate them and result in a very significant worsening of their quality of life. In our study, only 1 of 17 patients (less than 6%) experienced PD after the omission of adjuvant systemic therapy. In this case, the patient refused the treatment, although there was a strong clinical indication.

Data from cumulative incidence functions in our study highlight that, although survival remains high, PD affects a significant proportion of patients independently of mortality. In practice, it might be beneficial to manage PD to improve long-term patients’ outcomes, as a substantial portion of patients experienced PD despite remaining alive. So, interventions aiming to extend progression-free intervals may have a role in enhancing quality of life. It follows that a careful assessment of the risk/benefit ratio must guide the treatment process in this setting of patients, in addition to the patient’s preferences.

Therefore, after more than 8 years from the beginning of our study, about half of the patients were still alive, with a median age of 90.5 years, further supporting the need for a careful plan of treatment in such patients. In fact, ultra-old patients are indeed frail, but sometimes with a not negligible life expectancy, quality of life has to be always considered in relation to different available therapeutic options. More recently, Lodi et al. published the results of a retrospective study about BC women observed during 16 years, categorized by age, including 1105 patients over 75 years of age [42]. They reported more favorable pathological characteristics in older patients with BC but also more advanced stages at the diagnosis. Their findings also support the de-escalation of the axilla surgery with the advice of screening prolongation when the life expectancy is estimated to be sufficient. Similarly to us, they found frequent delayed access to care among old patients.

In order to promote earlier access to care and consequently a diagnosis of less advanced BC and less aggressive treatment, dedicated screening but also recommendations for geriatricians, general practitioners, and older patients’ relatives are important to implement the examination of breast glands or specifically ask about breasts when not directly evaluable. The education of patients and their relatives would likely advance BC diagnoses in the elderly.

In our study, we observed a significant percentage of drop-outs. For many older patients, there seems to be a high possibility of changing residence (for example, moving to a nursing home or relocating to a different town or region to live closer to or with relatives). For other frail patients with greater mobility impairment, accessing the hospital could be difficult, resulting in stopping visits and specific controls (though not necessarily ET, which can be supervised by the general practitioner). Nevertheless, the inclusion of all patients enrolled in the analyses on death was crucial to avoid the high risk of overestimating OS. This highlights the role of registry linkage in capturing vital outcomes for patients lost to direct follow-up, ensuring a more complete picture of long-term mortality within the cohort.

### 4.4. Strengths and Limitations

To our knowledge, this is the first prospective study focused on BC patients older than 80 years. We collected data not only about BC characteristics and therapies but also secondary data, including polytherapy, geriatric scores, familial and personal cancer history, etc. The main limitation of our study was the small number of patients enrolled, which narrows selected statistical analyses, for example, for competing risks. Moreover, the high number of drop-outs led to a lack of specific data about the cause of death, especially for patients who did not progress from BC. Furthermore, the percentage of general and specific drug toxicities could be underestimated due to missing data from patients lost to follow-up. Even though the relatively small number of patients did not allow us to address some specific statistical goals, our data could be useful for a future expansion of real-world data collection and meta-analyses.

### 4.5. Future Perspectives and Translational Aspects

As is well known, prospective studies, especially those involving prolonged follow-up, risk becoming outdated due to the rapid evolution of novel therapies. Therefore, larger, multicentric studies should be conducted to assess, in a shorter timeframe, how the approval and implementation of new therapies for breast cancer may impact outcomes in very elderly patients. As life expectancy continues to increase, statistical data indicate a concurrent rise in cancer diagnoses among the elderly. Thus, enhancing the focus on geriatric oncology is essential. Including older patients in clinical trials is paramount—not only to generate evidence for optimal treatment strategies tailored to this frail population but also to ensure the concurrent maintenance of quality of life and functional independence. This holistic approach will help address the unique challenges faced by elderly patients, ultimately improving both clinical outcomes and their overall well-being.

## 5. Conclusions

Treating and monitoring older breast cancer (BC) patients represent unique challenges due to the heterogeneity of this population. Older patients often have multiple comorbidities, functional limitations, and varying degrees of frailty, which can influence treatment choices and outcomes. The underrepresentation of elderly patients, particularly those over 80 years, in clinical trials often limits the generalizability of research findings.

We conducted a prospective, monocentric observational study on patients diagnosed with BC at the age of 80 and older. After more than 8 years from the beginning of our study, about half of the patients were still alive, with a median age of 90.5 years.

In our cohort, surgery seems to favorably impact patient outcome, while omitting endocrine adjuvant therapy does not significantly worsen the prognosis, except for higher-risk BC. BC care for ultra-older patients is currently relevant and will become even more significant due to the ever-increasing life expectancy.

Future research should prioritize the development of personalized treatment protocols that incorporate comprehensive geriatric assessments and quality-of-life metrics. In particular, larger, multicentric studies are essential to validate existing real-life findings and investigate the impact of emerging therapies in this age group. Collecting more data—ideally through prospective studies, but also via retrospective analyses of large databases—will provide a deeper understanding of the unique characteristics of elderly patients, facilitating the design of tailored, evidence-based treatment strategies.

## Figures and Tables

**Table 1:** Demographic and clinical characteristics of the 88 patients.

Characteristic	Total *n*	Women *n*
**Overall *n* (%)**	88 (100.0)	86 (100.0)
**Age at diagnosis (years)**		
Mean (SD)	84.5 (3.9)	84.5 (3.9)
Median (Q1, Q3)	83.5 (81.8, 87.0)	83.0 (81.3, 87.0)
Min–Max	80.0–101.0	80.0–101.0
**Biological sex, *n* (%)**		
Male	2 (2.3)	0
Female	86 (97.7)	86 (100.0)
**Autonomous, *n* (%)**		
No	15 (17.0)	15 (17.4)
Yes	72 (81.8)	70 (81.4)
Missing	1 (1.1)	1 (1.2)
**G8 score**		
Mean (SD)	12.5 (2.7)	12.6 (2.6)
Median (Q1, Q3)	13 (10.4, 15.0)	13.0 (10.5, 15.0)
Min–Max	5.5–16.0	5.5–16.0
**CCI score**		
Mean (SD)	2.0 (1.6)	1.9 (1.5)
Median (Q1, Q3)	2 (1, 3)	2 (1, 3)
Min–Max	0–7	0–7
**Delayed access to care, *n* (%)**		
No	63 (71.6)	62 (72.1)
Yes	24 (27.3)	23 (26.7)
Not available	1 (1.1)	1 (1.2)
**Presentation, *n* (%)**		
Not advanced	74 (84.1)	73 (84.9)
Advanced	14 (15.9)	13 (15.1)
**Previous (metachronous) BC, *n* (%)**		
No	72 (81.8)	71 (82.6)
Yes	16 (18.2)	15 (17.4)
**Other cancers in personal history, *n* (%)**		
No	71 (80.7)	71 (82.6)
Yes	17 (19.3)	15 (17.4)
Oncohematologic	4 (4.5)	4 (4.6)
Gastrointestinal	3 (3.4)	3 (3.5)
Lung	2 (2.3)	1 (1.2)
Bladder	2 (2.3)	2 (2.3)
Kidney	2 (2.3)	1 (1.2)
Endometrium	2 (2.3)	2 (2.3)
Cervix	1 (1.1)	1 (1.2)
Melanoma	1 (1.1)	1 (1.2)
**Concomitant pharmacotherapy, *n* (%)**		
Antihypertensives	65 (73.9)	65 (75.6)
Antiplatelets	36 (40.9)	34 (39.5)
Drugs/supplements for osteoporosis	29 (32.9)	29 (33.7)
CNS drugs	23 (26.1)	23 (26.7)
Levothyroxine	15 (17.0)	15 (17.4)
Antidiabetics	14 (15.9)	14 (16.3)
Anticoagulants	4 (4.5)	4 (4.6)
Not available/unreliable	4 (4.5)	4 (4.6)

Acronyms: n, number of cases; CNS, central nervous system.

**Table 2:** Diagnostic, surgical procedures and tumor characteristics of 91 neoplasms in 88 patients.

Characteristics	Total *n* (%)	Women *n* (%)
**Breast surgery, total**	91	89
No	28 (30.8)	27 (30.3)
Yes	63 (69.2)	62 (69.7)
Mastectomy	15 (16.5)	15 (16.9)
Quadrantectomy	45 (49.4)	45 (50.6)
Lumpectomy	3 (3.3)	2 (2.2)
**Lymph node surgery, total**	91	89
No	58 (63.7)	56 (62.9)
Yes	33 (36.3)	33 (37.1)
Sentinel lymph node biopsy	23 (25.3)	23 (25.9)
Axillary lymph node dissection	10 (11.0)	10 (11.2)
**Side, total**	91	89
Right	42 (46.2)	40 (44.9)
Left	49 (53.8)	49 (55.1)
**Size (mm)**		
Mean (SD)	21.9 (16.7)	21.7 (16.8)
Median (Q1, Q3)	18 (12, 30)	17.5 (12, 26)
Min–Max	4–105	4–105
**T stage, total**	81	80
T1	42 (51.9)	42 (52.5)
T2	22 (27.2)	22 (27.5)
T3	4 (4.9)	4 (5.0)
T4	13 (16.0)	12 (15.0)
**Presentation, total**	88	86
Not advanced	74 (84.1)	73 (84.9)
N0	22 (29.7)	22 (30.1)
N+	10 (13.5)	10 (13.7)
Nx	42 (56.8)	41 (56.2)
Advanced	14 (15.9)	13 (15.1)
Bone metastases	7 (8.0)	6 (7.0)
Lung metastases	4 (4.5)	3 (3.5)
Other	8 (9.1)	8 (9.3)
**Histotype, total**	91	89
No special type	55 (60.4)	53 (59.6)
Lobular	15 (16.5)	15 (16.8)
Other type	21 (23.1)	21 (23.6)
**Grade, total**	91	89
1	8 (8.8)	8 (9.0)
2	53 (58.2)	52 (58.4)
3	30 (33.0)	29 (32.6)
**LVI, total**	63	62
No	43 (68.3)	43 (69.4)
Yes	20 (31.7)	19 (30.6)
Focal	13 (20.6)	13 (21.0)
Extensive	7 (11.1)	6 (9.7)
**ER/PR, total**	91	89
Negative	9 (9.9)	9 (10.1)
Positive	82 (90.1)	80 (89.9)
**HER2, total**	91	89
Negative	82 (90.1)	80 (89.9)
Positive	9 (9.9)	9 (10.1)
**Ki-67, total**	91	89
Negative (<20%)	46 (50.5)	46 (51.7)
Positive (≥20%)	45 (49.5)	43 (48.3)
**Molecular type, total**	91	89
Luminal A like	38 (41.7)	38 (42.7)
Luminal B like	37 (40.7)	35 (39.3)
Luminal B-HER2+	7 (7.7)	7 (7.9)
HER2	2 (2.2)	2 (2.2)
Triple negative	7 (7.7)	7 (7.9)

Acronyms: n, number of cases; ER, estrogen receptor; PR, progesterone receptor; HER2, human epidermal growth factor receptor 2.

**Table 3:** Characteristics of 17 patients not receiving adjuvant systemic therapies.

Case	Age (yrs)	Histotype	Stage	Molecular Subtype	CCI	G8	Predict Score *	Additional OS Benefit **	PD	OS (Months)
1	81	Apocrine	T2Nx	TN	1	8	61%/78%	CT: 5.5%	N	104
2	93	NST	T1cNx	LA	3	13	NA	NA	N	30
3	90	NST	T1aNx	TN	5	10	NA	NA	N	37
4	87	NST	T1cNx	LB	2	13	NA	NA	N	49
5	81	NST	T1aN0 (sn)	LB-H+	4	16	76%/78%	ET/CT/T: 0.5%/0.4%/0.2%	N	88
6	88	NST	T1cNx	LA	1	14	NA	NA	N	69
7	86	Papillary	T2N0 (sn)	TN	3	14	NA	NA	N	88
8	82	NST	T2N0 (sn)	LA	1	16	73%/76%	ET: 1%	N	83
9	80	Mucinous	T1cN0 (sn)	LA	5	14	79%/80%	ET: 0.4%	N	82
10	83	Lobular	T2N3a	TN	2	11	27%/73%	CT: 11% ^¶^	Y	16
11	85	Mucinous	T1cNx	LA	1	9.5	66%/67%	ET: 0.6%	N	82
12	81	NST	T1cN0 (sn)	LA	2	15	77%/78%	ET: 0.3%	N	78
13	83	NST	T2Nx	LA	3	10.5	69%/73%	ET: 1.5%	N	78
14	89	Lobular	T1bNx	LA	1	12	NA	NA	N	76
15	81	Lobular	T1cN0 (sn)	LA	0	13	71%/73%	ET: 0.8%	N	81
16	82	Lobular	T1cNx	LA	7	15	73%/76%	ET: 0.8%	N	67
17	82	Lobular	T1cNx	LA	2	8	74%/76%	ET: 0.7%	N	48

Acronyms: NST, no special type; sn, sentinel; TN, triple negative; LA, luminal; LB, luminal B; LB-H+, luminal B-HER2 positive; NA, not available; CT, chemotherapy; ET, endocrine therapy; T, trastuzumab; OS, overall survival; PD, progressive disease; N, no; Y, yes.* Predict score: OS after only surgery, w/o systemic therapy/OS if deaths from BC were excluded. Not available (NA) for older than 85 years. ** Five years—OS additional benefit with systemic therapy. ^¶^ This patient was treated with loco-regional radiotherapy but refused CT.

## Data Availability

The original contributions presented in this study are included in the article/Supplementary Materials. Further inquiries can be directed to the corresponding author.

## Supplementary Materials

The following supporting information can be downloaded at: https://www.mdpi.com/article/10.3390/cancers16244142/s1, Figure S1. Kaplan-Meier curves (solid lines) for overall survival (a) and progression-free survival (b) over a 5-year (60 months) follow-up period evaluated only in female participants. Gray shaded areas represent the 95% confidence intervals for each survival estimate, while vertical segments (|) represent censored patients.
