# Supplementary material for: A Prospective Monocentric Study of Invasive Breast Carcinoma Diagnosed at 80 Years and Older: Survival Outcomes and Peculiar Challenges
Source: Cancers (Basel). 2024 Dec 12;16(24):4142. doi: 10.3390/cancers16244142 (PMC11674549; doi:10.3390/cancers16244142)
Supplement: Supplementary file 1 [file cancers-16-04142-s001.zip › cancers-3340848-supplementary.pdf]

Figure S1

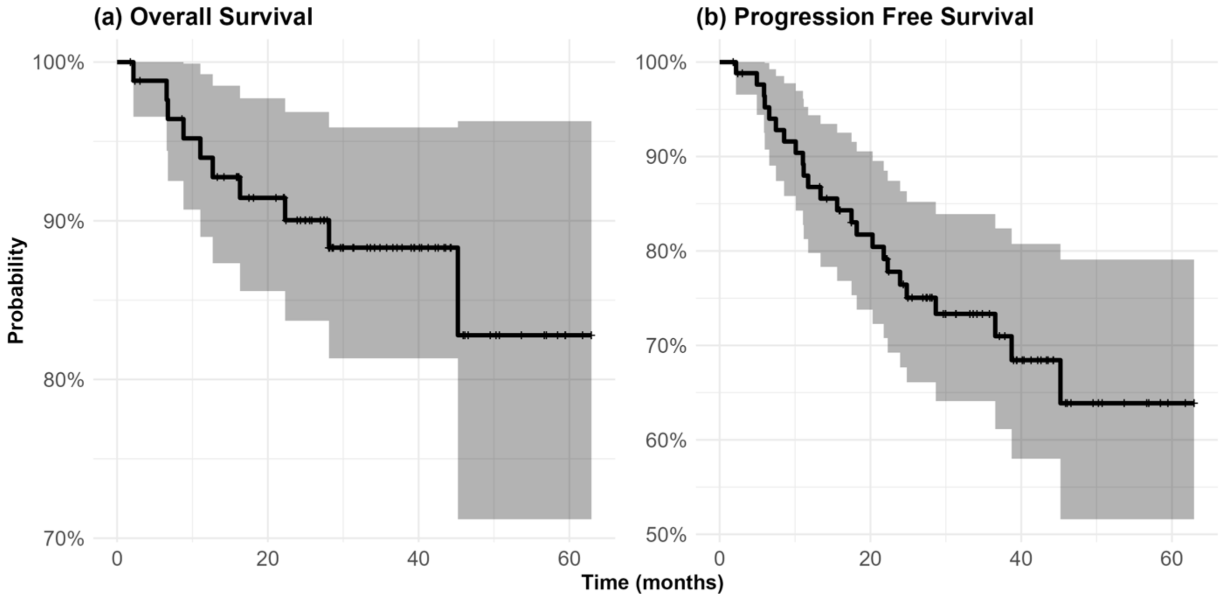

Figure S1. Kaplan-Meier curves (solid lines) for overall survival (a) and progression-free survival (b) over a 5-year (60 months) follow-up period evaluated only in female participants. Gray shaded areas represent the 95% confidence intervals for each survival estimate, while vertical segments (|) represent censored patients.
